# Identification of seven-coordinate Ln^III^ ions in a Ln^III^[15-MC_Fe_^III^_N(shi)_-5](OAc)_2_Cl species crystallized from methanol and pyridine

**DOI:** 10.1007/s10870-021-00900-6

**Published:** 2021-08-21

**Authors:** Elizabeth S. Biros, Cassandra L. Ward, Matthew J. Allen, Jacob C. Lutter

**Affiliations:** a Department of Chemistry, Wayne State University, 5101 Cass Avenue, Detroit, MI, 48202, USA; b Lumingen Instrument Center, Wayne State University, 5101 Cass Avenue, Detroit, MI. 48202, USA

**Keywords:** Iron, Lanthanides, Metallacrown, Self-assembly, Seven-coordinate complexes

## Abstract

The title metallacrown (MC) complexes Ln^III^[15-MC_Fe^III^N(shi)_-5](OAc)_2_CI(C_5_H_5_N)_6_ (**Ln1**), where OAc^−^ is acetate, shi^3−^ is salicylhydroximate, and Ln = Gd and Dy, were synthesized via a self-assembly reaction in methanol and pyridine. Single crystals were grown using slow evaporation and characterized using X-ray diffraction. Seven-coordinate capped octahedron geometries were observed for the lanthanide ion in both complexes, which is uncommon for trivalent lanthanide species. The 15-MC-5 is a ruffled metallacrown archetype similar to previously reported mixed-valent manganese metallacrowns.

## Graphical Abstract

The title metallacrown (MC) complexes Ln^III^[15-MC_Fe^III^N(shi)_-5](OAc)_2_Cl(C_5_H_5_N)_6_ (**Ln1**), where OAc^−^ is acetate, shi^3−^ is salicylhydroximate, and Ln = Gd and Dy, contains seven-coordinate capped octahedron geometries for the lanthanide ion in both complexes, which is uncommon for trivalent lanthanide species.

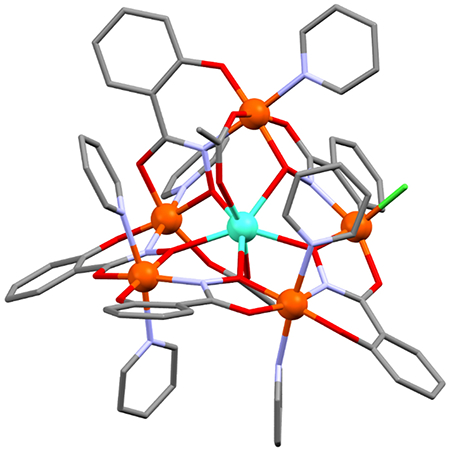

## Introduction

Trivalent lanthanide ions have gathered attention thanks to unique photophysical and magnetic properties inherent to their 4f valence electronic structure. The fact that the valence 4f orbitals are shielded from the ligand field by fully occupied 5s and 5p orbitals means that there is little bonding interaction between lanthanide 4f orbitals and ligand-based orbitals [1]. Therefore, spin-orbit coupling is unquenched and the electronic structure is protected from ligand-field effects. For photophysical applications, these properties lead to characteristic emissions unique to each trivalent lanthanide, as well as long luminescence lifetimes arising from the Laporte-forbidden nature of 4f–4f transitions. For magnetic applications, unquenched spin-orbit coupling leads to significant magnetoanisotropy that is attractive for single-molecule magnets, quantum computing, and spintronic devices [2–6]. In the case of gadolinium(III), which is an isotropic 4f^7^ ion, applications as contrast agents for magnetic resonance imaging [7] and magnetocoolents are common [8–15]. Although the ligand field does not have as strong of an effect on trivalent lanthanide ions as is observed in transition metal complexes, coordination geometry is important to consider when working with trivalent lanthanides—for example, ligand fields contribute to the barriers for magnetization relaxation [16]. For the most part, eight- and nine-coordinate geometries are observed for trivalent lanthanide ions; however, there has been a push to discover seven-coordinate complexes of lanthanides because their relatively asymmetric ligand field relieves some of the barrier from the Laporte-forbidden transitions compared to the more commonly observed eight- and nine-coordinate complexes [17]. The low symmetry associated with seven-coordinate trivalent lanthanides enables lanthanide luminescence with shorter lifetimes and more readily accessible native excitation compared to more symmetrical complexes [18–20]. This asymmetry is also useful for molecular magnetism relative to more symmetrical complexes because asymmetry results in more inherent magnetoanisotropy and different ligand-field effects that influence the barrier to magnetization relaxation [21–23].

**Figure 1.**
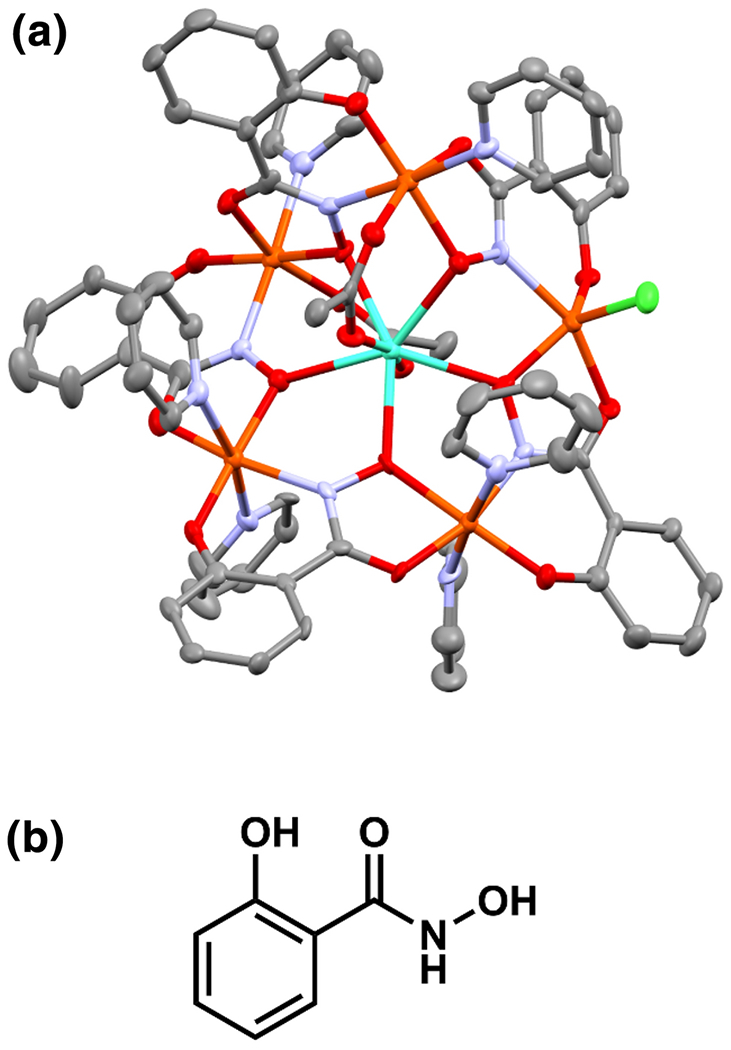
a) Representation of **Gd1** from single-crystal X-ray diffraction data. Teal = gadolinium, orange = iron, green = chlorine, red = oxygen, blue = nitrogen, and gray = carbon. Solvent molecules and hydrogen atoms have been removed for clarity. Thermal ellipsoids are drawn at 50% certainty. b) Schematic of the salicylhydroxamic acid parent ligand (H_3_shi).

A system that can be modified to induce different coordination environments upon trivalent lanthanides is the metallacrowns. Metallacrowns (MCs) were first introduced by Pecoraro and Lah in 1989 [24], and since then, many metallacrowns have been studied with potential impact on applications involving magnetism and luminescence. Metallacrowns are metallamacrocyclic coordination complexes with structures analogous to organic crown ethers, in which C–C–O repeating units are replaced by M–N–O repeating units. These units are typically comprised of a 3d-transition or main-group metals and hydroximate ligands. Many examples of 3d-4f heterobimetallic metallacrowns feature transition metals in the ring as well as a lanthanide ion within the central cavity [25–27]. Some metallacrowns use optically transparent metal ions, such as zinc(II) or gallium(III), in the ring to sensitize lanthanide-based luminescence [28–36], and others use paramagnetic metal ions, such as manganese(III) or iron(III), to explore molecular magnetism [37–40]. However, among these examples of metallacrown structures there are no reports of an encapsulated seven-coordinate trivalent lanthanide. The title compound, Ln^III^[15-MC_Fe^III^N(shi)_-5](OAc)_2_Cl(C_5_H_5_N)_6_ (**Ln1**, Figure 1), is the first example of a metallacrown that fits this description.

## Experimental

### Materials

Iron(III) chloride hexahydrate (98%, reagent grade chunks), dysprosium(III) nitrate hydrate (99.9%), gadolinium(III) nitrate hexahydrate (99.9%), and pyridine (99%, ACS reagent) were purchased from Sigma-Aldrich. Salicylhydroxamic acid (99%) was purchased from Acros Organics. Glacial acetic acid and methanol (ACS regent) were purchased from EMD Chemicals. Iron(III) chloride hexahydrate chunks were pulverized using a mortar and pestle, then stored in a desiccator. All other reagents were used as received.

Elemental analysis was performed by Midwest Microlabs, Inc.

### Synthetic Methods

General synthesis of Ln^III^[15-MC_Fe^III^N(shi)_-5](OAc)_2_Cl(C_5_H_5_N)_6_ (**Ln1**), Ln = Gd and Dy

To a flask was added Gd(NO_3_)_3_·6H_2_O (0.0564 g, 0.125 mmol, 1 equiv) or Dy(NO_3_)_3_·H_2_O (0.0436 g, 0.125 mmol, 1 equiv), FeCl_3_·6H_2_O (0.1689 g, 0.6250 mmol, 5 equiv), and H_3shi_ (0.0957 g, 0.625 mmol, 5 equiv). These solids were dissolved in a solution of methanol (10 mL) and pyridine (2 mL). To the resulting solution was added glacial acetic acid (0.100 mL, 1.75 mmol, 14 equiv). The resulting reddish-brown reaction mixture was stirred for 1 h, then gravity filtered using #1 Whatman filter paper. Slow evaporation of the mother liquor yielded dark red block crystals in about 1 week.

Gd^III^[15-MC_Fe^III^N(shi)_-5](OAc)_2_Cl(C_5_H_5_N)_6_·2C_5_H_5_N·3CH_3_OH·H_2_O (**Gd1**): Synthetic yield = 33% based on gadolinium nitrate hexahydrate. Elemental analysis of GdFe_5_C_82_H_80_N_13_O_23_Cl [MM = 2087.54 g/mol] observed (calculated): %C 47.40 (47.18); %H 3.48 (3.86); %N 8.53 (8.72). Unit Cell: a = 14.1017(14) Å, b = 16.0027(15) Å, c = 17.4534(17) Å, α = 89.674(3)°, β = 79.831(3)°, γ = 74.278(3)°, V = 3728.02 Å^3^.

Dy^III^[15-MC_Fe^III^N(shi)_-5](OAc)_2_Cl(C_5_H_5_N)_6_·C_5_H_5_N·CH_3_OH·H_2_O (**Dy1**): Synthetic yield = 41% based on dysprosium nitrate hydrate. Elemental analysis of DyFe_5_C_75_H_67_N_12_O_21_Cl [MM = 1949.60 g/mol] observed (calculated): %C 46.47 (46.21); %H 3.26 (3.46); %N 8.66 (8.62). Unit Cell: a = 14.1019(9) Å, b = 15.9679(10) Å, c = 17.4781(11) Å, α = 89.862(2)°, β = 79.712(2)°, γ = 74.702(2)°, V = 3730.97 Å^3^.

### X-ray Crystallography

Dark red blocks of **Gd1** were grown from a solution of methanol and pyridine at 22 °C. A crystal of dimensions 0.207 mm × 0.202 mm × 0.135 mm was mounted using a MicroMount (MiTeGen) with paratone oil (Parabar 10312, Hampton Research) onto a Bruker X8 Apex-II CCD-based X-ray diffractometer using a Mo sealed tube anode (λ = 0.71073 Å) equipped with a graphite monochromator. The X-ray intensities were measured at 100(2) K using an Oxford 800 Cryostream with a detector placed at a distance of 40 mm from the crystal. Using Apex3 v2019.11-0, the crystal contained two domains that were separated using Cell Now. The intensities were integrated using SAINT V8.40b and TWINABS-2012/1 was used for absorption correction. The integration yielded a total of 25530 reflections to a maximum 2θ value of 50.34° of which 13302 were independent and 14348 were greater than 2σ(I). The final cell constants were based on xyz centroids of 3497 reflections above 10σ(I). Analysis of the data showed negligible decay during collection. The structure was solved with SHELXT [41] and refined using SHELXL (version 2018/3) [42], using the space group $\text{P}\bar{1}$ with Z = 2 for the formula C_74.25_H_56_Fe_5_N_12_O_19.25_GdCl. All nonhydrogen atoms were refined anisotropically with the hydrogen atoms placed in idealized positions. The structure was refined as a nonmerohedral twin using HKLF5 format with a final BASF of 0.3459. Full matrix least-squares refinement based on F^2^ converged at R_1_ = 0.0812 and wR_2_ = 0.1726 [based on I > 2σ(I)] and R_1_ = 0.1682 and wR_2_ = 0.2144 for all data. More details are listed in Table 1, and the refined structure is available in CIF format.

Dark red blocks of **Dy1** were grown from a solution of methanol and pyridine at 22 °C. A crystal of dimensions 0.22 mm × 0.15 mm × 0.06 mm was mounted using a MicroMount (MiTeGen) with paratone oil (Parabar 10312, Hampton Research) onto a Bruker X8 Apex-II CCD-based X-ray diffractometer using a Mo sealed tube anode (λ = 0.71073 Å) equipped with a graphite monochromator. The X-ray intensities were measured at 100(2) K using an Oxford 800 Cryostream with a detector placed at a distance of 40 mm from the crystal. Using Apex3 v2019.11-0, the crystal contained two domains that were separated using Cell Now. The intensities were integrated using SAINT V8.40b and TWINABS-2012/1 was used for absorption correction. The integration yielded a total of 13823 reflections to a maximum 2θ value of 50.99° of which 16029 were independent and 7385 were greater than 2σ(I). The final cell constants were based on xyz centroids of 8352 reflections above 10σ(I). Analysis of the data showed negligible decay during collection. The structure was solved with SHELXT [41] and refined using SHELXL (version 2018/3) [42], using the space group $\text{P}\bar{1}$ with Z = 2 for the formula C_74_H_62_Fe_5_N_11_O_19.75_DyCl. All nonhydrogen atoms were refined anisotropically with the hydrogen atoms placed in idealized positions. The structure was a nonmerohedral twin and refined using HKLF5 format with a final BASF of 0.17671. Full matrix least-squares refinement based on F^2^ converged at R_1_ = 0.0795 and wR_2_ = 0.1379 [based on I > 2σ(I)] and R_1_ = 0.1947 and wR_2_ = 0.1764 for all data. More details are listed in Table 1, and the refined structure is available in CIF format.

## Results and Discussion

Both **Gd1** and **Dy1** were synthesized via self-assembly in methanol and pyridine. Both compounds are 15-MC-5 complexes using the metallacrown analogy first described by Pecoraro and Lah in 1989 (Figure S1, Supplemental Information) [24]. The metallacrown moiety is comprised of five shi^3−^, five Fe^3+^ ions, one Ln^3+^ ion, two OAc^−^ ions that bridge the central Ln^3+^ to the iron MC ring, and six coordinated pyridine molecules. The metallamacrocycle is comprised of five Fe^3+^ and five shi^3−^, resulting in an overall neutral charge. A trivalent lanthanide is encapsulated in the center of the ring, and the charge of the lanthanide is balanced by two OAc^−^ ligands that span the central ion and a ring iron as well as one Cl^−^ ion that is bound to another ring iron.

**Figure 2.**
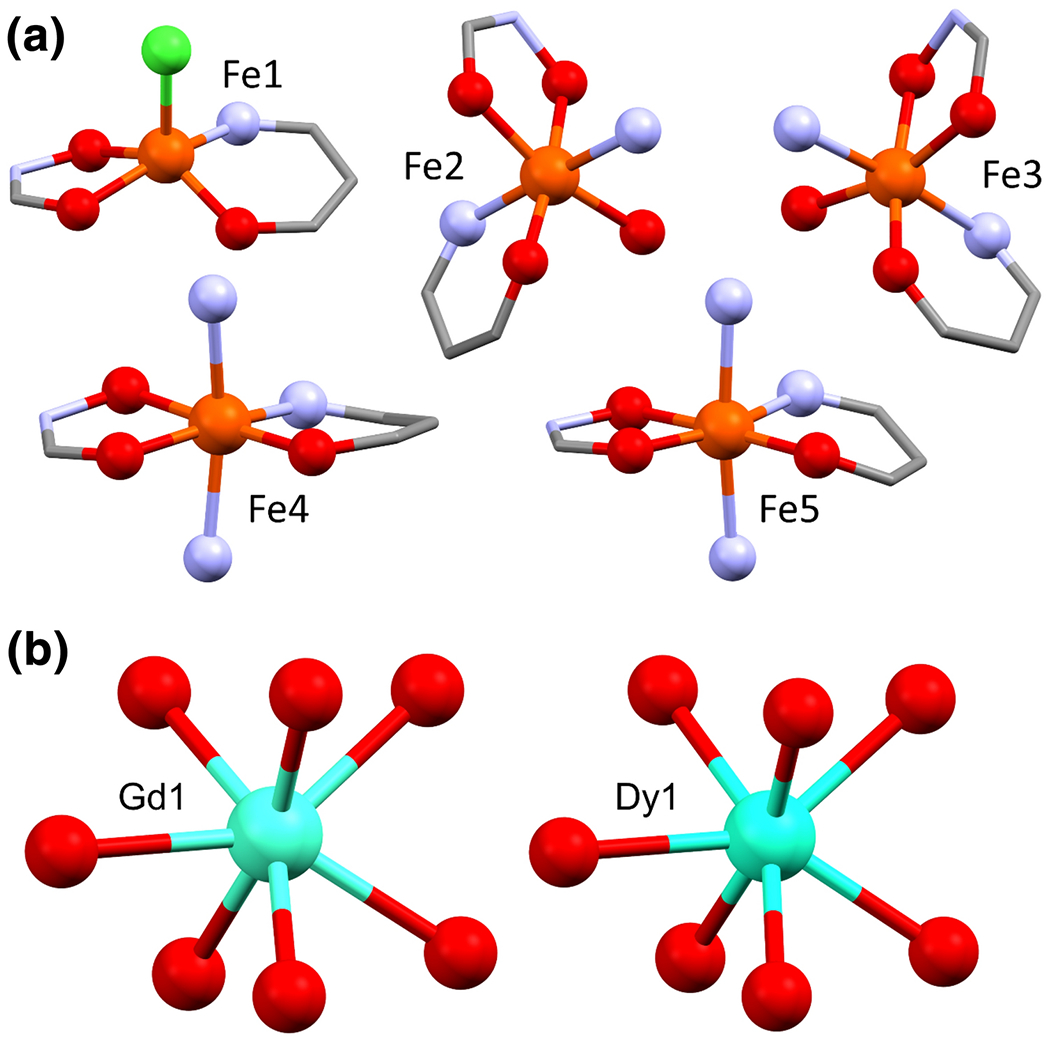
a) Coordination geometries for each iron center in **Gd1**, the five- and six-membered chelate rings of shi^3−^ are shown to represent the whole shi^3−^ moiety, pyridine and acetate ligands are abbreviated as a single nitrogen or oxygen, respectively. b) Coordination geometry of the Ln ion in each compound. Aqua = gadolinium or dysprosium, orange = iron, green = chlorine, blue = nitrogen, red = oxygen, and grey = carbon.

Geometric information is summarized in Tables 2 and 3 for each of the metal centers, and isolated representations of the iron coordination environments in **Gd1** are displayed in Figure 2 and coordination geometries were confirmed using SHAPE (v2.1) analysis.[43] One iron center (Fe1) is in a five-coordinate square-based pyramid geometry and bound by two shi^3−^ and one Cl^−^. Addison τ values were about 0.39 for both compounds, supporting a square-pyramid geometry assignment [44]. Two iron centers in each compound (Fe4 and Fe5) are in six-coordinate octahedral geometries and bound to two shi^3−^ and two pyridine ligands. The shi^3−^ are both in the equatorial positions of the octahedron and have compressed Fe-O bond lengths for the bond connecting the iron to a phenolic oxygen in shi^3−^, and the pyridine molecules are in the axial positions with elongated Fe–N bond lengths compared to the average bond length of each iron center (Tables 2 and 3). These distortions are likely due to geometric constraints from the 15-MC-5 metallacycle. The final two iron centers (Fe2 and Fe3) are in six-coordinate octahedral geometries and are bound to two shi^3−^, one oxygen of OAc^−^, and one pyridine molecule in a propeller conformation. There is a compressed Fe–O bond between the iron and a phenolic oxygen in shi^3−^ as well as an elongated Fe–N bond from the iron to the pyridine molecule compared to the average bond length of each iron center (Tables 2 and 3). These distortions are also likely a result of geometric constraints from the 15-MC-5 metallacycle structure. These propeller-type iron centers are also chiral, where one is Δ and the other is ∧. Bond-valence sums support the observation that each iron ion is in the +3 oxidation state [45, 46].

The Ln ions are each seven-coordinate and in geometries that most closely resemble capped octahedrons according to SHAPE (v2.1) analysis.[43] Each Ln ion is bound to each of the five oxime oxygens of the shi^3−^ and one oxygen from each of the two bridging OAc^−^ ligands (Figure 2). For **Gd1**, the Gd–O bonds to the OAc^−^ are compressed to about 2.29 Å and two of the Gd–O bonds to oxime oxygens are slightly elongated at 2.376 and 2.359 Å, compared to the average Gd–O bond length of 2.336 Å. **Dy1** also displays compression of the Dy–O bonds to about 2.26 Å with the OAc^−^ oxygens and elongation of one Dy–O bond to 2.356 Å with an oxime oxygen in shi^3−^, compared to the average Dy–O bond length of 2.313 Å. The metallacrown cavity radius was calculated using the method outlined by Pecoraro and coworkers for copper 15-MC-5 compounds [47]. **Gd1** had a cavity radius of 1.135 Å, and **Dy1** had a cavity radius of 1.115 Å. These slight differences in average bond length, bond distortions, and cavity radii are likely due to the difference in ionic radius between Gd^3+^ and Dy^3+^ [48]. Bond-valence sums support the observation that each Ln ion is in the +3 oxidation state [49].

**Figure 3.**
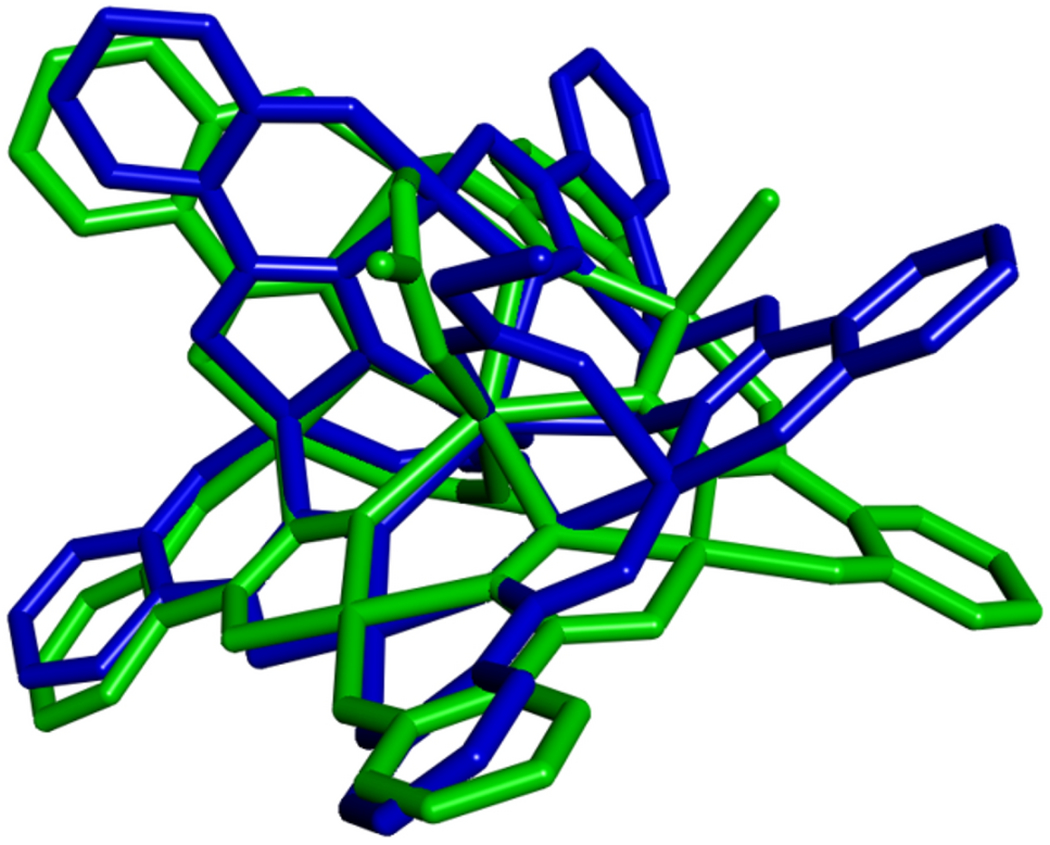
Overlay of representations of X-ray diffraction data for **Gd1** (green) and a reported Mn^II^[15-MC_Mn^III^N(shi)_-5](C_3_H_5_O_2_)_2_(C_4_H_6_N_2_)_4_(C_3_H_7_NO)_2_ compound (blue) [55]. The overlay was generated using Discovery Studio 2021.[56]

The ruffled structure of **Ln1** arises from the combination of equatorial and propeller octahedral iron centers in the metallacrown ring. For example, **Gd1** follows the cycle of square pyramidal, Δ propeller, ∧ propeller, equatorial, equatorial; where the MC ring is bent by the Δ propeller and the ∧ propeller to complete the metallamacrocycle as a ruffled structure. This motif is common to Mn^II^[15-MC_Mn^III^N(shi)_-5] compounds [50–55]. Overlay of **Gd1** with one example of these manganese mixed-valence 15-MC-5s, that uses propanoate instead of acetate to bridge the central metal and a combination of N-methyl imidazole/N,N-dimethylformamide rather than pyridine to complete the ring metal coordination spheres, [55] demonstrates similarity between the two structures (Figure 3). These similarities include the capped-octahedron geometry of the central metal ion, and differ only by the positioning of the carboxylate ligands and the presence or absence of Cl^−^ that causes one shi^3−^ to be in a different orientation.

**Figure 4.**
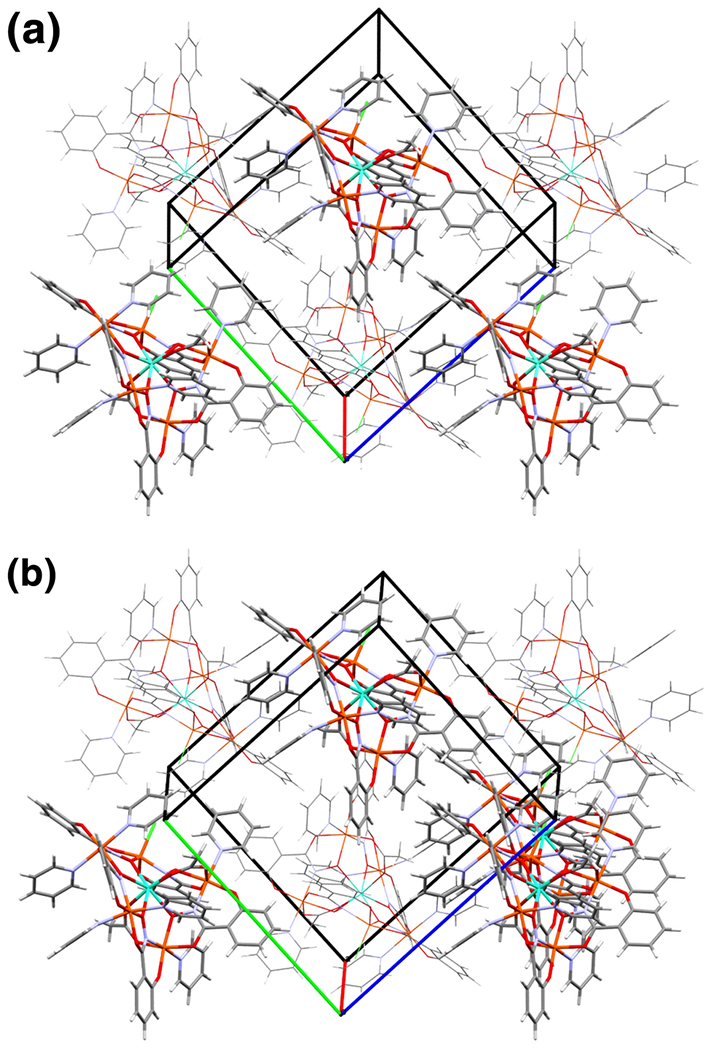
Packing diagrams from crystallographic data for **Gd1** (a) and **Dy1** (b) are essentially identical, though unit cells arbitrarily lie on different lattice points for each structure. Aqua = gadolinium or dysprosium, orange = iron, green = chlorine, blue = nitrogen, red = oxygen, gray = carbon, white = hydrogen. Solvent atoms are omitted for clarity.

Both **Gd1** and **Dy1** have a nearly identical packing motif, which is to be expected in the case of changing only the lanthanide ion that is encapsulated (Figure 4). There are two cases of intermolecular π–π stacking, including; the shi^3−^ containing the ring of C9 through C14 and the pyridine containing the ring of N6 and C40 through C44, and the shi^3−^ containing the ring of C15 through C21 and the pyridine containing the ring of N7 and C45 through C49. In addition, two examples of H–π stacking are observed where H42 of a pyridine interacts with the ring containing C30 through C35 of a shi^3−^, and H57 of a pyridine interacts with the ring containing C23 through C28 of a shi^3−^. Lastly, four D–H–A intermolecular hydrogen bonds are observed; two that involve a shi^3−^ C–H bond to a carbonyl oxygen or phenolic oxygen of another shi^3−^, and two that involve a pyridine C–H interacting with Cl1 (Table 4 and 5). The same intermolecular hydrogen bonds are observed for both analogs, and the geometric parameters of these interactions are nearly the same values.

## Conclusions

Two iron 15-MC-5 compounds are reported with gadolinium(III) or dysprosium(III) bound in the central cavity. These metallacrowns adopt ruffled motifs that are similar to other mixed-valent manganese metallacrown complexes in which the MC ring is bent by the Δ propeller and the ∧ propeller to complete the metallamacrocycle as a ruffled structure. The trivalent lanthanide ions are encapsulated in the metallacrowns in uncommon seven-coordinate capped-octahedron geometries. This seven-coordinate geometry has not yet been observed in a metallacrown compound, and could lead to interesting magnetic and lanthanide-based luminescence properties in the future.

## Supplementary Material

1744594_Sup_info

## Figures and Tables

**Table 1.** Crystallographic parameters for **Ln1**.

Compound	**Gd1** (2083355)	**Dy1** (2083356)
Chemical Formula	C_74.25_H_62_Fe_5_N_12_O_19.25_GdCl	C_74_H_61_Fe_5_N_12_O_19.75_DyCl
Formula Weight	1902.30 g/mol	1911.55 g/mol
Crystal System, Space Group	Triclinic, $\text{P}\bar{1}$ (No. 2)	Triclinic, $\text{P}\bar{1}$ (No. 2)
T	100(2) K	100(2) K
a	14.1017(14) Å	14.1019(9) Å
b	16.0027(15) Å	15.9676(10) Å
c	17.4543(17) Å	17.4781(11) Å
α	89.674(3)°	89.862(2)°
β	79.831(3)°	79.712(2)°
γ	74.278(3)°	74.702(2)°
Volume	3728.0(6) Å^3^	3731.0(4) Å^3^
λ	0.71073 Å	0.71073 Å
ρ_calc_	1.695 g/cm^3^	1.702 g/cm^3^
Z	2	2
μ	1.941 mm^−1^	2.053 mm^−1^
F(000)	1913	1920
range	1.186° to 25.169°	1.185° to 25.494°
Limiting Indices	−16 < h < 16	−16 < h < 17
	−19 < k < 19	−19 < k < 19
	0 < I < 20	0 < I < 21
Reflections collected/unique	25530/14348	13823/7385
Completeness to θ	99.4%	99.9%
No. of Data/Restraints/Params	25530/1031/12	13823/1030/57
GooF on F^2^	0.985	1.029
^a^R_1_	0.0812 [I > 2σ(I)]; 0.1682 [all data]	0.0796 [I > 2σ(I)]; 0.1948 [all data]
^b^wR_2_	0.1727 [I > 2σ(I)]; 0.2148 [all data]	0.1379 [I > 2σ(I)]; 0.1764 [all data]
Largest Diff. Peak, Hole	1.919 e Å^−3^, −1.477 e Å^−3^	1.499 e Å^−3^, −1.431 e Å^−3^

a R_1_ = Σ(‖F_o_|-|F_c_‖)/Σ|F_o_|

b wR_2_ = [Σ[w(F_o_^2^ − F_c_^2^)^2^]/Σ[w(F_o_)^2^]]^1/2^; w = 1/[σ^2^(F_o_^2^) + (*mp*)^2^ + *np*]; *p* = [max(F_o_^2^,0) + 2F_c_^2^]/3 (*m* and *n* are constants); σ = [Σ[w(F_o_^2^ − F_c_^2^)^2^/(*n* − *p*)]^1/2^

**Table 2.** Geometric information for metal centers in **Gd1**.

Metal	Avg. Bond Length (Å)	Coord. Number	Shape	CShM^1^	BVS	Additional Info
Gd1	2.336	7	capped octahedron	0.79847	3.101	----
Fe1	2.020	5	square pyramid	1.73866	3.084	τ = 0.3895
Fe2	2.030	6	octahedron	0.79770	3.115	Δ propeller
Fe3	2.039	6	octahedron	1.03182	3.061	∧ propeller
Fe4	2.066	6	octahedron	1.40048	2.950	equatorial
Fe5	2.056	6	octahedron	0.98982	3.037	equatorial

1 The smallest CShM compared to ideal positions for coordination geometry

**Table 3.** Geometric information for metal centers in **Dy1**.

Metal	Avg. Bond Length (Å)	Coord. Number	Shape	CShM^1^	BVS	Additional Info
Dy1	2.313	7	capped octahedron	0.77961	3.098	----
Fe1	2.020	5	square pyramid	1.67781	3.080	τ = 0.3863
Fe2	2.026	6	octahedron	0.75189	3.152	Δ propeller
Fe3	2.037	6	octahedron	1.10793	3.091	∧ propeller
Fe4	2.064	6	octahedron	1.37055	2.965	equatorial
Fe5	2.048	6	octahedron	0.98386	3.092	equatorial

1 The smallest CShM compared to ideal positions for coordination geometry

**Table 4.** Geometric information for D–H–A hydrogen bonds observed in **Gd1**.

Hydrogen Bond	D–H Distance (Å)	H–A Distance (Å)	D–A Distance (Å)	D–H–A Angle (°)
C13–H13 … O2	0.95	2.40	3.241	146.9
C27–H27 … O3	0.95	2.64	3.552	160.2
C41–H41 … Cl1	0.95	2.96	3.804	149.3
C63–H63 … Cl1	0.95	2.75	3.534	140.8

**Table 5.** Geometric information for D–H–A hydrogen bonds observed in **Dy1**.

Hydrogen Bond	D–H Distance (Å)	H–A Distance (Å)	D–A Distance (Å)	D–H–A Angle (°)
C13–H13 … O2	0.95	2.41	3.255	148.0
C27–H27 … O3	0.95	2.63	3.542	161.1
C41–H41 … Cl1	0.95	2.95	3.818	151.9
C63–H63 … Cl1	0.95	2.79	3.561	138.7

## Data Availability

Single-crystal diffraction data are available in CIF format free of charge at www.ccdc.cam.ac.uk using deposition numbers 2083355 and 2083356.
