# Supplementary material for: Identification of seven-coordinate LnIII ions in a LnIII[15-MCFeIIIN(shi)-5](OAc)2Cl species crystallized from methanol and pyridine
Source: J Chem Crystallogr. Author manuscript; Available in PMC 2023 Jun 1. (PMC9122301; doi:10.1007/s10870-021-00900-6)
Supplement: 1744594_Sup_info [file NIHMS1744594-supplement-1744594_Sup_info.docx]

**Identification of seven-coordinate Ln^III^ ions in a Ln^III^[15-MC_Fe_^III^_N(shi)_-5](OAc)_2_Cl species crystallized from methanol and pyridine**

Elizabeth S. Biros,^a^ Cassandra L. Ward,^b^ Matthew J. Allen,^a^ and Jacob C. Lutter^a,^*

^a^ Department of Chemistry, Wayne State University, 5101 Cass Avenue, Detroit, MI, 48202, USA

^b^ Lumingen Instrument Center, Wayne State University, 5101 Cass Avenue, Detroit, MI. 48202, USA

*Corresponding Email: jclutter@umich.edu

Supplemental Information

Table of Contents

**Figure S1.** Representation of the 15-MC-5 motif in **Gd1**……..…………...…………………………………….2

**Figure S2.** ORTEP representation of **Gd1**………………………...…………………………………………...…2

**Figure S3.** ORTEP representation of **Dy1**……………………………………………………………………..…3

**Table S1.** Selected bond distances for **Gd1** metal centers………………………..…………………………………………….3

**Table S2.** Selected bond angles for **Gd1** metal centers…………………………………………………………4

**Table S3.** Selected bond distances for **Dy1** metal centers………………………..…………………………….6

**Table S4.** Selected bond angles for **Dy1** metal centers…………………………………………………………7

SHAPE analysis for metal ion coordination environments………………………………………………..……..9


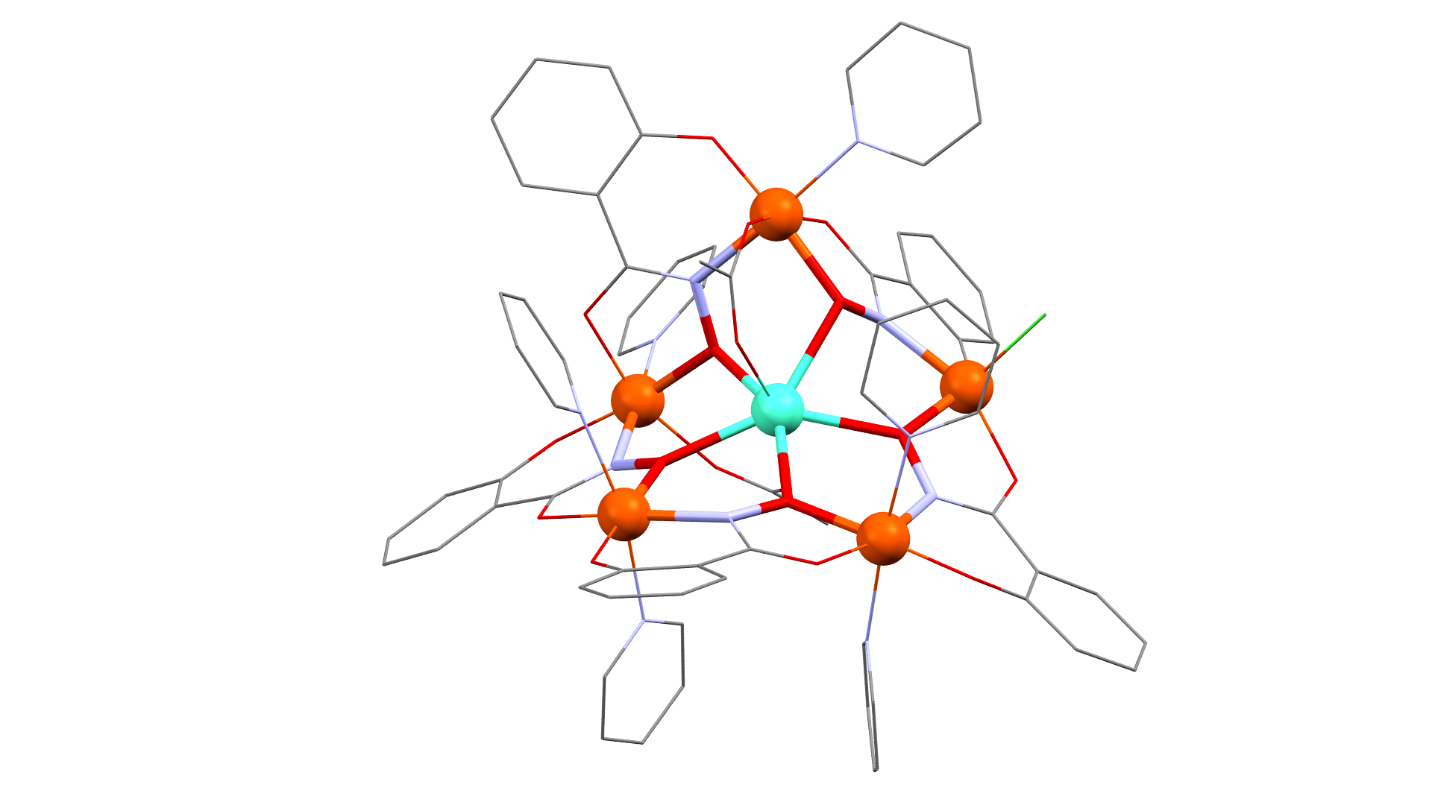


**Figure S1.** Representation from X-ray diffraction data of **Gd1** with the 15-MC-5 motif highlighted. Teal = gadolinium, orange = iron, green = chlorine, blue = nitrogen, red = oxygen, and grey = carbon. Hydrogen atoms and solvent molecules are omitted for clarity.


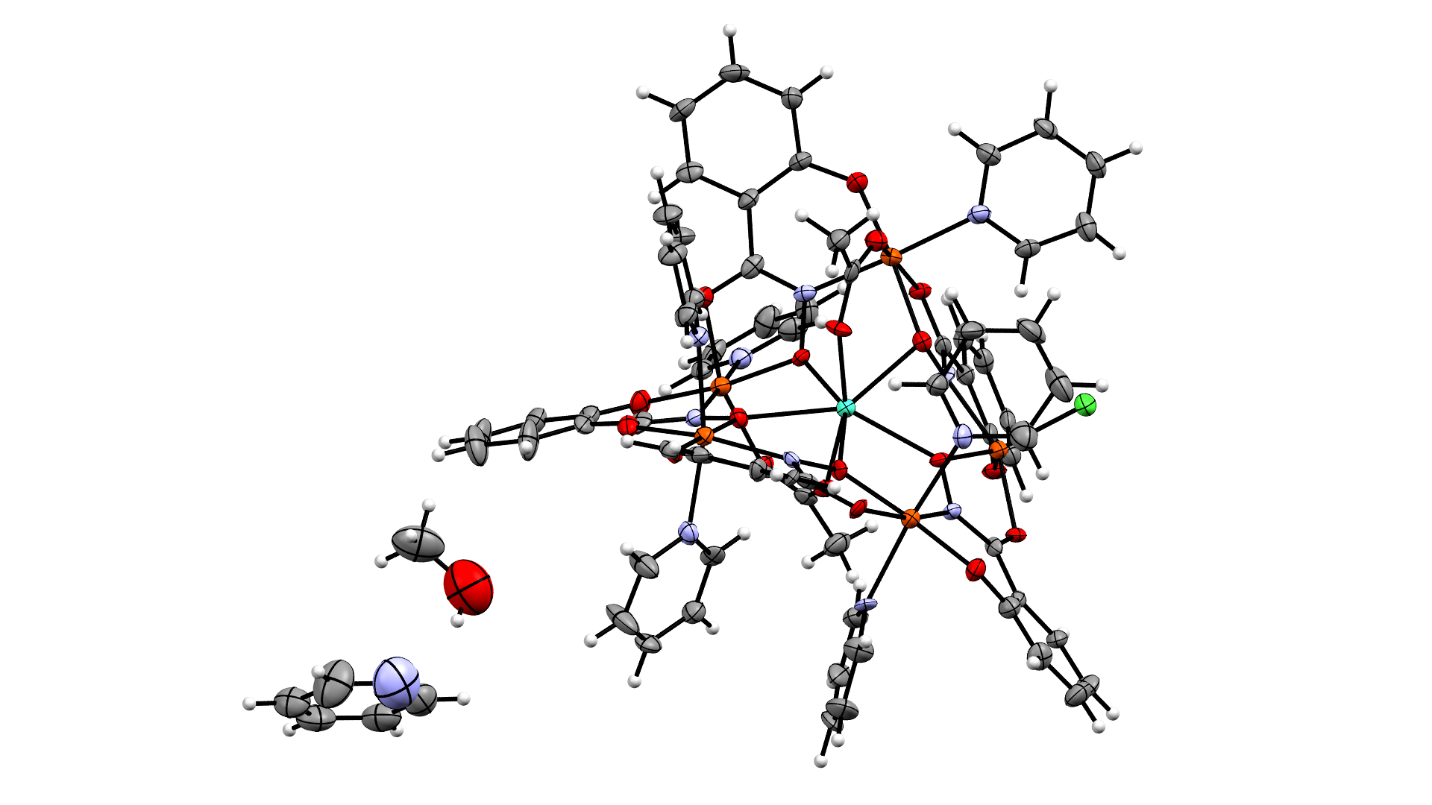


**Figure S2.** ORTEP representation of **Gd1**. Teal = Gd, orange = iron, green = chlorine, blue = nitrogen, red = oxygen, gray = carbon. Thermal ellipsoids are drawn at 50% probability.


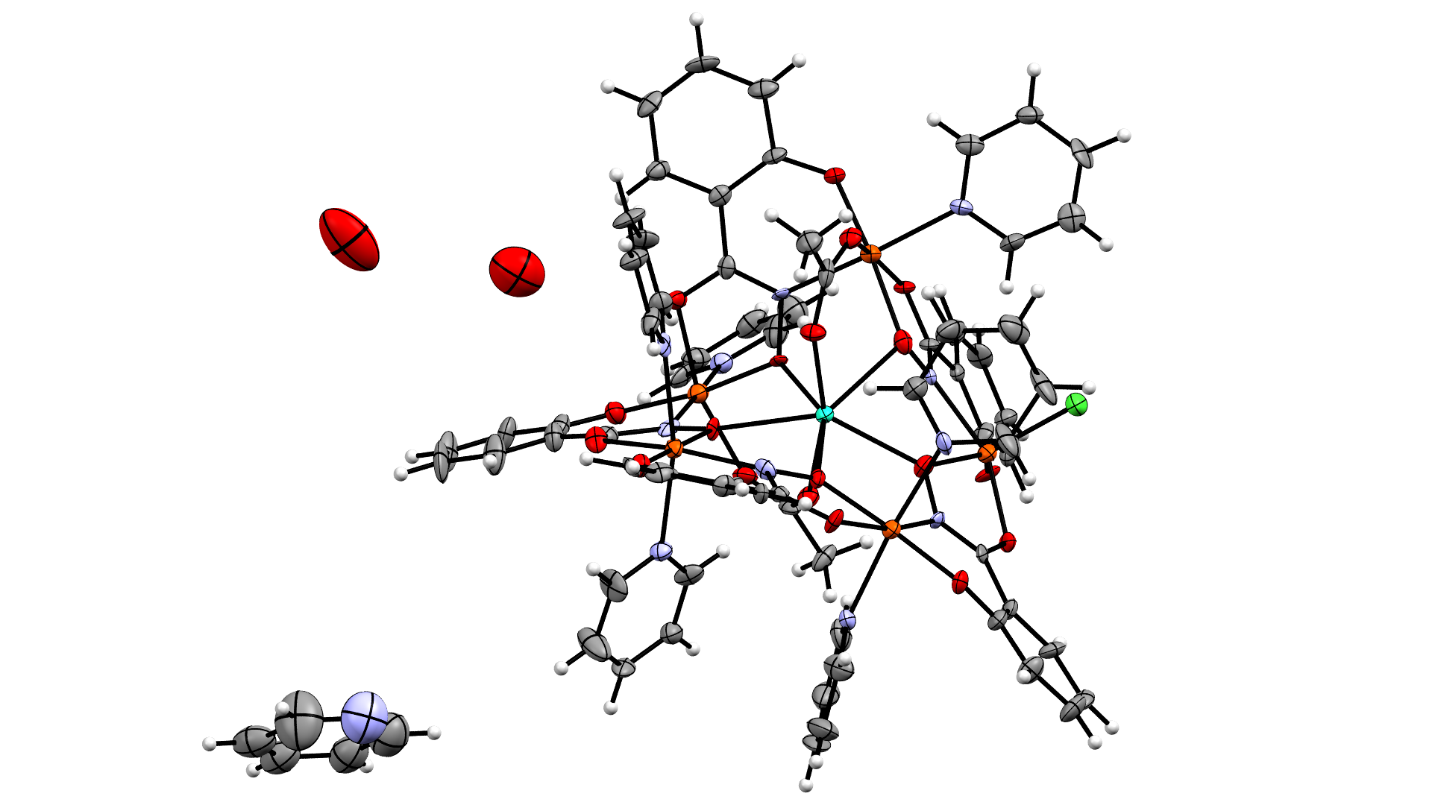


**Figure S3.** ORTEP representation of **Dy1**. Teal = Dy, orange = iron, green = chlorine, blue = nitrogen, red = oxygen, gray = carbon. Thermal ellipsoids are drawn at 50% probability.

**Table S1.** Selected bond distances for **Gd1** metal centers.

| **Bond** | **Bond Length/Å** |
| --- | --- |
| Gd(1)–O(16) | 2.292(7) |
| Gd(1)–O(18) | 2.294(8) |
| Gd(1)–O(10) | 2.344(7) |
| Gd(1)–O(1) | 2.344(7) |
| Gd(1)–O(4) | 2.345(7) |
| Gd(1)–O(13) | 2.359(7) |
| Gd(1)–O(7) | 2.376(7) |
| Fe(1)–O(3) | 1.867(8) |
| Fe(1)–O(13) | 1.968(7) |
| Fe(1)–O(14) | 1.992(7) |
| Fe(1)–N(1) | 2.034(9) |
| Fe(1)–Cl(1) | 2.237(3) |
| Fe(2)–O(6) | 1.886(7) |
| Fe(2)–O(2) | 2.009(7) |
| Fe(2)–O(17) | 2.025(8) |
| Fe(2)–O(1) | 2.029(7) |
| Fe(2)–N(2) | 2.075(9) |
| Fe(2)–N(6) | 2.158(9) |
| Fe(3)–O(9) | 1.878(8) |
| Fe(3)–O(4) | 1.996(7) |
| Fe(3)–O(5) | 2.021(8) |
| Fe(3)–O(19) | 2.056(8) |
| Fe(3)–N(3) | 2.097(9) |
| Fe(3)–N(7) | 2.185(10) |
| Fe(4)–O(12) | 1.888(7) |
| Fe(4)–O(7) | 1.992(7) |
| Fe(4)–O(8) | 1.994(7) |
| Fe(4)–N(4) | 2.086(9) |
| Fe(4)–N(8) | 2.214(10) |
| Fe(4)–N(9) | 2.223(10) |
| Fe(5)–O(15) | 1.892(7) |
| Fe(5)–O(11) | 1.943(7) |
| Fe(5)–N(5) | 2.033(9) |
| Fe(5)–O(10) | 2.044(7) |
| Fe(5)–N(10) | 2.198(10) |
| Fe(5)–N(11) | 2.226(9) |

**Table S2.** Selected bond angles for **Gd1** metal centers.

| **Bonds** | **Bond Angle/^o^** |
| --- | --- |
| O(16)–Gd(1)–O(18) | 165.0(3) |
| O(16)–Gd(1)–O(10) | 84.7(3) |
| O(18)–Gd(1)–O(10) | 92.7(3) |
| O(16)–Gd(1)–O(1) | 77.8(3) |
| O(18)–Gd(1)–O(1) | 114.1(3) |
| O(10)–Gd(1)–O(1) | 132.1(2) |
| O(16)–Gd(1)–O(4) | 96.7(3) |
| O(18)–Gd(1)–O(4) | 78.6(2) |
| O(10)–Gd(1)–O(4) | 151.6(2) |
| O(1)–Gd(1)–O(4) | 75.3(2) |
| O(16)–Gd(1)–O(13) | 113.9(3) |
| O(18)–Gd(1)–O(13) | 79.5(2) |
| O(10)–Gd(1)–O(13) | 75.7(2) |
| O(1)–Gd(1)–O(13) | 71.6(2) |
| O(4)–Gd(1)–O(13) | 127.9(2) |
| O(16)–Gd(1)–O(7) | 84.0(3) |
| O(18)–Gd(1)–O(7) | 81.0(3) |
| O(10)–Gd(1)–O(7) | 75.5(2) |
| O(1)–Gd(1)–O(7) | 144.2(2) |
| O(4)–Gd(1)–O(7) | 76.4(2) |
| O(13)–Gd(1)–O(7) | 144.2(2) |
| O(3)–Fe(1)–O(13) | 133.0(3) |
| O(3)–Fe(1)–O(14) | 94.4(3) |
| O(13)–Fe(1)–O(14) | 77.9(3) |
| O(3)–Fe(1)–N(1) | 88.2(4) |
| O(13)–Fe(1)–N(1) | 83.1(3) |
| O(14)–Fe(1)–N(1) | 156.4(3) |
| O(3)–Fe(1)–Cl(1) | 114.3(3) |
| O(13)–Fe(1)–Cl(1) | 112.6(2) |
| O(14)–Fe(1)–Cl(1) | 99.3(2) |
| N(1)–Fe(1)–Cl(1) | 101.0(3) |
| O(6)–Fe(2)–O(2) | 100.0(3) |
| O(6)–Fe(2)–O(17) | 94.8(3) |
| O(2)–Fe(2)–O(17) | 164.8(3) |
| O(6)–Fe(2)–O(1) | 174.3(3) |
| O(2)–Fe(2)–O(1) | 76.9(3) |
| O(17)–Fe(2)–O(1) | 88.6(3) |
| O(6)–Fe(2)–N(2) | 85.5(3) |
| O(2)–Fe(2)–N(2) | 89.7(3) |
| O(17)–Fe(2)–N(2) | 94.8(3) |
| O(1)–Fe(2)–N(2) | 89.6(3) |
| O(6)–Fe(2)–N(6) | 91.3(3) |
| O(2)–Fe(2)–N(6) | 90.2(3) |
| O(17)–Fe(2)–N(6) | 86.2(3) |
| O(1)–Fe(2)–N(6) | 93.6(3) |
| N(2)–Fe(2)–N(6) | 176.7(3) |
| O(9)–Fe(3)–O(4) | 170.9(3) |
| O(9)–Fe(3)–O(5) | 94.1(3) |
| O(4)–Fe(3)–O(5) | 77.6(3) |
| O(9)–Fe(3)–O(19) | 100.2(3) |
| O(4)–Fe(3)–O(19) | 88.6(3) |
| O(5)–Fe(3)–O(19) | 163.1(3) |
| O(9)–Fe(3)–N(3) | 86.5(3) |
| O(4)–Fe(3)–N(3) | 90.6(3) |
| O(5)–Fe(3)–N(3) | 94.6(3) |
| O(19)–Fe(3)–N(3) | 95.2(3) |
| O(9)–Fe(3)–N(7) | 88.6(4) |
| O(4)–Fe(3)–N(7) | 94.4(3) |
| O(5)–Fe(3)–N(7) | 87.6(3) |
| O(19)–Fe(3)–N(7) | 83.8(3) |
| N(3)–Fe(3)–N(7) | 174.8(4) |
| O(12)–Fe(4)–O(7) | 177.5(3) |
| O(12)–Fe(4)–O(8) | 103.4(3) |
| O(7)–Fe(4)–O(8) | 78.9(3) |
| O(12)–Fe(4)–N(4) | 89.2(3) |
| O(7)–Fe(4)–N(4) | 88.7(3) |
| O(8)–Fe(4)–N(4) | 163.6(3) |
| O(12)–Fe(4)–N(8) | 89.7(4) |
| O(7)–Fe(4)–N(8) | 89.6(3) |
| O(8)–Fe(4)–N(8) | 86.9(3) |
| N(4)–Fe(4)–N(8) | 103.8(3) |
| O(12)–Fe(4)–N(9) | 89.9(3) |
| O(7)–Fe(4)–N(9) | 91.3(3) |
| O(8)–Fe(4)–N(9) | 80.7(3) |
| N(4)–Fe(4)–N(9) | 89.0(3) |
| N(8)–Fe(4)–N(9) | 167.2(3) |
| O(15)–Fe(5)–O(11) | 105.8(3) |
| O(15)–Fe(5)–N(5) | 88.4(3) |
| O(11)–Fe(5)–N(5) | 165.6(3) |
| O(15)–Fe(5)–O(10) | 173.6(3) |
| O(11)–Fe(5)–O(10) | 78.4(3) |
| N(5)–Fe(5)–O(10) | 87.8(3) |
| O(15)–Fe(5)–N(10) | 91.2(3) |
| O(11)–Fe(5)–N(10) | 91.3(3) |
| N(5)–Fe(5)–N(10) | 85.4(4) |
| O(10)–Fe(5)–N(10) | 93.6(3) |
| O(15)–Fe(5)–N(11) | 85.4(3) |
| O(11)–Fe(5)–N(11) | 94.4(3) |
| N(5)–Fe(5)–N(11) | 89.5(4) |
| O(10)–Fe(5)–N(11) | 89.4(3) |
| N(10)–Fe(5)–N(11) | 174.0(4) |

**Table S3.** Selected bond distances for **Dy1** metal centers.

| **Bond** | **Bond Length/Å** |
| --- | --- |
| Dy(1)–O(19) | 2.257(7) |
| Dy(1)–O(16) | 2.270(7) |
| Dy(1)–O(1) | 2.320(7) |
| Dy(1)–O(7) | 2.325(7) |
| Dy(1)–O(10) | 2.328(7) |
| Dy(1)–O(4) | 2.335(7) |
| Dy(1)–O(13) | 2.356(7) |
| Fe(1)–O(3) | 1.881(7) |
| Fe(1)–O(14) | 1.990(8) |
| Fe(1)–O(13) | 2.001(7) |
| Fe(1)–N(1) | 2.085(9) |
| Fe(1)–N(6) | 2.197(9) |
| Fe(1)–N(7) | 2.231(9) |
| Fe(2)–O(12) | 1.883(7) |
| Fe(2)–O(17) | 2.011(7) |
| Fe(2)–O(8) | 2.011(7) |
| Fe(2)–O(7) | 2.017(8) |
| Fe(2)–N(4) | 2.074(9) |
| Fe(2)–N(10) | 2.159(9) |
| Fe(3)–O(6) | 1.894(7) |
| Fe(3)–O(2) | 1.953(7) |
| Fe(3)–N(2) | 2.010(9) |
| Fe(3)–O(1) | 2.026(7) |
| Fe(3)–N(8) | 2.192(9) |
| Fe(3)–N(9) | 2.214(9) |
| Fe(4)–O(9) | 1.870(7) |
| Fe(4)–O(4) | 1.983(7) |
| Fe(4)–O(5) | 1.989(7) |
| Fe(4)–N(3) | 2.022(8) |
| Fe(4)–Cl(1) | 2.235(3) |
| Fe(5)–O(15) | 1.863(8) |
| Fe(5)–O(10) | 1.981(7) |
| Fe(5)–O(11) | 2.032(7) |
| Fe(5)–O(18) | 2.049(8) |
| Fe(5)–N(5) | 2.110(9) |
| Fe(5)–N(11) | 2.187(9) |

**Table S4.** Selected bond angles for **Dy1** metal centers.

| **Bonds** | **Bond Angle/^o^** |
| --- | --- |
| O(19)–Dy(1)–O(16) | 165.4(3) |
| O(19)–Dy(1)–O(1) | 93.6(3) |
| O(16)–Dy(1)–O(1) | 84.2(3) |
| O(19)–Dy(1)–O(7) | 114.1(3) |
| O(16)–Dy(1)–O(7) | 77.4(3) |
| O(1)–Dy(1)–O(7) | 131.5(2) |
| O(19)–Dy(1)–O(10) | 79.4(3) |
| O(16)–Dy(1)–O(10) | 95.8(3) |
| O(1)–Dy(1)–O(10) | 152.0(2) |
| O(7)–Dy(1)–O(10) | 75.1(2) |
| O(19)–Dy(1)–O(4) | 79.2(3) |
| O(16)–Dy(1)–O(4) | 114.0(3) |
| O(1)–Dy(1)–O(4) | 75.4(2) |
| O(7)–Dy(1)–O(4) | 72.1(3) |
| O(10)–Dy(1)–O(4) | 128.6(2) |
| O(19)–Dy(1)–O(13) | 80.5(2) |
| O(16)–Dy(1)–O(13) | 84.9(3) |
| O(1)–Dy(1)–O(13) | 76.1(2) |
| O(7)–Dy(1)–O(13) | 144.2(3) |
| O(10)–Dy(1)–O(13) | 76.0(2) |
| O(4)–Dy(1)–O(13) | 143.6(2) |
| O(3)–Fe(1)–O(14) | 103.8(3) |
| O(3)–Fe(1)–O(13) | 177.7(3) |
| O(14)–Fe(1)–O(13) | 78.4(3) |
| O(3)–Fe(1)–N(1) | 88.8(3) |
| O(14)–Fe(1)–N(1) | 164.2(3) |
| O(13)–Fe(1)–N(1) | 89.1(3) |
| O(3)–Fe(1)–N(6) | 89.0(3) |
| O(14)–Fe(1)–N(6) | 86.8(3) |
| O(13)–Fe(1)–N(6) | 90.4(3) |
| N(1)–Fe(1)–N(6) | 103.0(3) |
| O(3)–Fe(1)–N(7) | 90.6(3) |
| O(14)–Fe(1)–N(7) | 80.6(3) |
| O(13)–Fe(1)–N(7) | 90.5(3) |
| N(1)–Fe(1)–N(7) | 90.0(3) |
| N(6)–Fe(1)–N(7) | 167.0(3) |
| O(12)–Fe(2)–O(17) | 94.3(3) |
| O(12)–Fe(2)–O(8) | 99.7(3) |
| O(17)–Fe(2)–O(8) | 165.5(3) |
| O(12)–Fe(2)–O(7) | 174.3(3) |
| O(17)–Fe(2)–O(7) | 88.7(3) |
| O(8)–Fe(2)–O(7) | 77.6(3) |
| O(12)–Fe(2)–N(4) | 86.2(3) |
| O(17)–Fe(2)–N(4) | 94.3(3) |
| O(8)–Fe(2)–N(4) | 90.1(3) |
| O(7)–Fe(2)–N(4) | 88.7(3) |
| O(12)–Fe(2)–N(10) | 90.8(3) |
| O(17)–Fe(2)–N(10) | 85.9(3) |
| O(8)–Fe(2)–N(10) | 90.4(3) |
| O(7)–Fe(2)–N(10) | 94.2(3) |
| N(4)–Fe(2)–N(10) | 177.0(3) |
| O(6)–Fe(3)–O(2) | 105.6(3) |
| O(6)–Fe(3)–N(2) | 88.1(3) |
| O(2)–Fe(3)–N(2) | 165.9(3) |
| O(6)–Fe(3)–O(1) | 174.1(3) |
| O(2)–Fe(3)–O(1) | 78.4(3) |
| N(2)–Fe(3)–O(1) | 88.1(3) |
| O(6)–Fe(3)–N(8) | 90.9(3) |
| O(2)–Fe(3)–N(8) | 90.6(3) |
| N(2)–Fe(3)–N(8) | 86.0(3) |
| O(1)–Fe(3)–N(8) | 93.4(3) |
| O(6)–Fe(3)–N(9) | 85.7(3) |
| O(2)–Fe(3)–N(9) | 94.9(3) |
| N(2)–Fe(3)–N(9) | 89.1(3) |
| O(1)–Fe(3)–N(9) | 89.8(3) |
| N(8)–Fe(3)–N(9) | 174.1(3) |
| O(9)–Fe(4)–O(4) | 133.8(3) |
| O(9)–Fe(4)–O(5) | 94.4(3) |
| O(4)–Fe(4)–O(5) | 78.1(3) |
| O(9)–Fe(4)–N(3) | 88.7(3) |
| O(4)–Fe(4)–N(3) | 83.3(3) |
| O(5)–Fe(4)–N(3) | 156.9(3) |
| O(9)–Fe(4)–Cl(1) | 113.9(3) |
| O(4)–Fe(4)–Cl(1) | 112.3(2) |
| O(5)–Fe(4)–Cl(1) | 98.7(2) |
| N(3)–Fe(4)–Cl(1) | 100.9(2) |
| O(15)–Fe(5)–O(10) | 170.2(3) |
| O(15)–Fe(5)–O(11) | 93.8(3) |
| O(10)–Fe(5)–O(11) | 77.4(3) |
| O(15)–Fe(5)–O(18) | 100.0(3) |
| O(10)–Fe(5)–O(18) | 89.4(3) |
| O(11)–Fe(5)–O(18) | 163.5(3) |
| O(15)–Fe(5)–N(5) | 86.6(3) |
| O(10)–Fe(5)–N(5) | 89.7(3) |
| O(11)–Fe(5)–N(5) | 95.0(3) |
| O(18)–Fe(5)–N(5) | 94.8(3) |
| O(15)–Fe(5)–N(11) | 88.3(4) |
| O(10)–Fe(5)–N(11) | 95.7(3) |
| O(11)–Fe(5)–N(11) | 87.5(3) |
| O(18)–Fe(5)–N(11) | 83.9(3) |
| N(5)–Fe(5)–N(11) | 174.4(4) |

**Table S5.** Shape Analysis for Dy1 in **Dy1**.

| **Shape** | **Shape Code** | **Point Group** | **CShM** |
| --- | --- | --- | --- |
| Heptagon | HP-7 | D_7h_ | 33.54843 |
| Hexagonal Pyramid | HPY-7 | C_6v_ | 20.90944 |
| Pentagonal Bipyramid | PBPY-7 | D_5h_ | 3.81455 |
| Capped Octahedron | COC-7 | C_3v_ | 2.05188 |
| Capped Trigonal Prism | CTPR-7 | C_2v_ | 0.77961 |
| Johnson Pentagonal Bipyramid | JPBPY-7 | D_5h_ | 7.04430 |
| Elongated Triangular Pyramid | JETPY-7 | C_3v_ | 20.95856 |

**Table S6.** Shape Analysis for Fe1 in **Dy1**.

| **Shape** | **Shape Code** | **Point Group** | | **CShM** | |
| --- | --- | --- | --- | --- | --- |
| Pentagon | PP-5 | | D_5h_ | | 32.40576 |
| Vacant Octahedron | vOC-5 | | C_4v_ | | 4.65931 |
| Trigonal Bipyramid | TBPY-5 | | D_3h_ | | 2.30290 |
| Square Pyramid | SPY-5 | | C_4v_ | | 1.67781 |
| Johnson Trigonal Bipyramid | JTBPY-5 | | C_5v_ | | 5.60962 |

**Table S7.** Shape Analysis for Fe2 in **Dy1**.

| **Shape** | **Shape Code** | **Point Group** | **CShM** |
| --- | --- | --- | --- |
| Hexagon | HP-6 | D_6h_ | 31.42955 |
| Pentagonal Pyramid | PPY-6 | C_5v_ | 25.34098 |
| Octahedron | OC-6 | O_h_ | 0.75198 |
| Trigonal Prism | TPR-6 | D_3h_ | 13.29236 |
| Johnson Pentagonal Pyramid | JPPY-6 | C_5v_ | 29.05744 |

**Table S8.** Shape Analysis for Fe3 in **Dy1**.

| **Shape** | **Shape Code** | **Point Group** | **CShM** |
| --- | --- | --- | --- |
| Hexagon | HP-6 | D_6h_ | 33.29598 |
| Pentagonal Pyramid | PPY-6 | C_5v_ | 24.45464 |
| Octahedron | OC-6 | O_h_ | 1.10793 |
| Trigonal Prism | TPR-6 | D_3h_ | 11.68004 |
| Johnson Pentagonal Pyramid | JPPY-6 | C_5v_ | 28.49053 |

**Table S7.** Shape Analysis for Fe4 in **Dy1**.

| **Shape** | **Shape Code** | **Point Group** | **CShM** |
| --- | --- | --- | --- |
| Hexagon | HP-6 | D_6h_ | 30.87600 |
| Pentagonal Pyramid | PPY-6 | C_5v_ | 24.58905 |
| Octahedron | OC-6 | O_h_ | 1.37055 |
| Trigonal Prism | TPR-6 | D_3h_ | 11.30645 |
| Johnson Pentagonal Pyramid | JPPY-6 | C_5v_ | 27.38279 |

**Table S7.** Shape Analysis for Fe5 in **Dy1**.

| **Shape** | **Shape Code** | **Point Group** | **CShM** |
| --- | --- | --- | --- |
| Hexagon | HP-6 | D_6h_ | 31.27614 |
| Pentagonal Pyramid | PPY-6 | C_5v_ | 24.56320 |
| Octahedron | OC-6 | O_h_ | 0.98386 |
| Trigonal Prism | TPR-6 | D_3h_ | 13.69324 |
| Johnson Pentagonal Pyramid | JPPY-6 | C_5v_ | 27.64947 |

**Table S8.** Shape Analysis for Gd1 in **Gd1**.

| **Shape** | **Shape Code** | **Point Group** | **CShM** |
| --- | --- | --- | --- |
| Heptagon | HP-7 | D_7h_ | 33.70545 |
| Hexagonal Pyramid | HPY-7 | C_6v_ | 21.00663 |
| Pentagonal Bipyramid | PBPY-7 | D_5h_ | 3.80226 |
| Capped Octahedron | COC-7 | C_3v_ | 1.92260 |
| Capped Trigonal Prism | CTPR-7 | C_2v_ | 0.79847 |
| Johnson Pentagonal Bipyramid | JPBPY-7 | D_5h_ | 7.09419 |
| Elongated Triangular Pyramid | JETPY-7 | C_3v_ | 20.79464 |

**Table S9.** Shape Analysis for Fe1 in **Gd1**.

| **Shape** | **Shape Code** | **Point Group** | | **CShM** | |
| --- | --- | --- | --- | --- | --- |
| Pentagon | PP-5 | | D_5h_ | | 32.56411 |
| Vacant Octahedron | vOC-5 | | C_4v_ | | 4.80824 |
| Trigonal Bipyramid | TBPY-5 | | D_3h_ | | 2.34702 |
| Square Pyramid | SPY-5 | | C_4v_ | | 1.73866 |
| Johnson Trigonal Bipyramid | JTBPY-5 | | C_5v_ | | 5.58406 |

**Table S10.** Shape Analysis for Fe2 in **Gd1**.

| **Shape** | **Shape Code** | **Point Group** | **CShM** |
| --- | --- | --- | --- |
| Hexagon | HP-6 | D_6h_ | 31.13844 |
| Pentagonal Pyramid | PPY-6 | C_5v_ | 25.27481 |
| Octahedron | OC-6 | O_h_ | 0.79770 |
| Trigonal Prism | TPR-6 | D_3h_ | 13.20850 |
| Johnson Pentagonal Pyramid | JPPY-6 | C_5v_ | 29.07860 |

**Table S11.** Shape Analysis for Fe3 in **Gd1**.

| **Shape** | **Shape Code** | **Point Group** | **CShM** |
| --- | --- | --- | --- |
| Hexagon | HP-6 | D_6h_ | 33.22937 |
| Pentagonal Pyramid | PPY-6 | C_5v_ | 24.67091 |
| Octahedron | OC-6 | O_h_ | 1.03182 |
| Trigonal Prism | TPR-6 | D_3h_ | 11.89635 |
| Johnson Pentagonal Pyramid | JPPY-6 | C_5v_ | 28.67885 |

**Table S12.** Shape Analysis for Fe4 in **Gd1**.

| **Shape** | **Shape Code** | **Point Group** | **CShM** |
| --- | --- | --- | --- |
| Hexagon | HP-6 | D_6h_ | 31.14274 |
| Pentagonal Pyramid | PPY-6 | C_5v_ | 24.58641 |
| Octahedron | OC-6 | O_h_ | 1.40048 |
| Trigonal Prism | TPR-6 | D_3h_ | 11.08104 |
| Johnson Pentagonal Pyramid | JPPY-6 | C_5v_ | 27.37201 |

**Table S13.** Shape Analysis for Fe5 in **Gd1**.

| **Shape** | **Shape Code** | **Point Group** | **CShM** |
| --- | --- | --- | --- |
| Hexagon | HP-6 | D_6h_ | 31.12556 |
| Pentagonal Pyramid | PPY-6 | C_5v_ | 24.58434 |
| Octahedron | OC-6 | O_h_ | 0.98982 |
| Trigonal Prism | TPR-6 | D_3h_ | 13.59641 |
| Johnson Pentagonal Pyramid | JPPY-6 | C_5v_ | 27.74600 |
